# Clinical trials provide *the* evidence critical for patient empowerment

**DOI:** 10.7448/IAS.16.1.18811

**Published:** 2013-08-19

**Authors:** Kevin M De Cock, Wafaa M El-Sadr

**Affiliations:** 1 Centers for Disease Control and Prevention, Atlanta, GA, USA; 2 ICAP at Columbia University, New York, NY, USA

In this issue of the Journal, Delva et al. discuss in a *Viewpoint* our Perspective article published in the *New England Journal of Medicine* in which we argue for the urgent need for a clinical trial on when to initiate antiretroviral therapy (ART) in HIV-infected patients in sub-Saharan Africa [1]. The authors posit that there is currently sufficient evidence to make informed decisions regarding this issue and consequently individual patients' autonomy should be the key factor in determining the timing for ART initiation.

As readers can review our Perspective article, we will not repeat arguments concerning the lack of definitive evidence to guide ART initiation, the limited evidence from observational studies nor the limitations of ongoing studies assessing timing for initiation of ART for African settings. Nonetheless, a few points that directly relate to the *Viewpoint* authors’ arguments are important to address.

With regard to the observational studies the authors cite as evidence in support of early initiation of ART, two of the articles do not provide relevant information to the question of early versus deferred ART [2, 3] and the other two are focused on the use of ART in individuals with early or acute HIV infection [4, 5]. Recently, Sabin et al. carefully reviewed the available observational studies related to early versus deferred ART initiation and highlighted the inconsistent estimates of benefit particularly with regard to mortality, the modest effect size noted with early ART use and the risk of confounding inherent to observational studies [6].

Another argument that the authors present is that the use of early ART is associated with a protective effect against tuberculosis. However, it is important to note that the majority of available evidence thus far is based on the use of ART in patients with advanced HIV disease [7]. In addition, the meta-analysis cited by the *Viewpoint's* authors included a limited number of individuals who initiated ART in the higher CD4+ cell count strata, mostly derived from observational studies [8]. HPTN 052, the sole randomized study included in the former meta-analysis with relevant patient population, did not demonstrate a statistically significant reduction in the incidence of pulmonary tuberculosis, even though the control group initiated ART well below the advised CD4+ cell count threshold of 350 cell/mm^3^ [9]. We believe that clarification and quantification of the preventive benefit of early ART on tuberculosis incidence is a strong argument for the clinical trial we propose.

The authors argue that diversity in guidelines and practice internationally mainly reflects differences in financial resources available across regions, rather than the absence of definitive evidence. We disagree with this position; the attitudes and practices in the United Kingdom and other parts of Europe, for example, where therapy upon early diagnosis is not recommended, differ from those in the United States, but the reasons relate to different interpretations of the evidence and different approaches to care, rather than primarily financial considerations [10].

The authors state that clinical trials are most appropriate for investigating efficacy and safety of new regimens, and are not of relevance to strategy questions that can inform guidelines. This overlooks the many clinical trials that compared various strategies rather than new drug regimens and that have had profound impact on guidelines and clinical practice. Relevant examples include HPTN 052, the SMART study, the SAPIT tuberculosis trial and the CIPRA 001 Haiti trial [9, 11–13]. In the realm of HIV prevention, large-scale randomized clinical trials are planned to provide a definitive answer as to whether the efficacy of treatment as prevention demonstrated in HPTN 052 will be realized at a population level [14]. We believe that a parallel trial is needed to answer the question with regard to the balance of benefits and risks for those with early HIV disease who would need to be treated [15].

There are compelling reasons to believe that early use of ART may best prevent adverse consequences of HIV infection. However, guidelines should be based as much as possible on firm evidence rather than expert opinion, especially when applied to millions of people and necessitating billions of dollars of funding. While the World Health Organization has released new guidelines that recommend initiation of ART in adults at CD4+ cell count of <500 cell/mm^3^, the guideline document itself states that “further research is required to determine more fully the clinical benefits and disadvantages of earlier ART initiation” [16]. Thus, even as guidelines change based on limited evidence, this should not stop the quest for definitive answers to critical clinical questions.

The *Viewpoint* pits patient autonomy versus clinical trials as reflected in the title. In contrast, we believe that clinical trials are necessary to provide the best quality evidence that would inform clinical guidelines and enable clinicians and patients to make informed decisions. We are struck that the individual approach advocated by the authors, with each patient/clinician making an independent decision based on incomplete evidence, is the antithesis of the public health approach promoted by the World Health Organization which has been the foundation of ART scale-up and perhaps the key factor allowing for successful access to ART to more than 9 million people globally, the majority in Africa [17].

Health is both a personal right and responsibility, and no one should take medicines against his or her will. However, consistent advice based on the strongest evidence is an essential requirement for program implementation and assurance of accountability, and is fundamental to informed and empowered patients.
